# Mapping of QTL for kernel abortion caused by *in vivo* haploid induction in maize (*Zea mays* L.)

**DOI:** 10.1371/journal.pone.0228411

**Published:** 2020-02-05

**Authors:** Yanzhi Qu, Penghao Wu, Jiaojiao Ren, Zonghua Liu, Jihua Tang, Thomas Lübberstedt, Haochuan Li, Shaojiang Chen

**Affiliations:** 1 College of Agronomy, National Key Laboratory of Wheat and Maize Crop Science, Collaborative Innovation Center of Henan Grain Crops, Henan Agricultural University, Zhengzhou, China; 2 National Maize Improvement Center, China Agricultural University, Beijing, China; 3 College of Agronomy, Xinjiang Agricultural University, Urumuqi, China; 4 Department of Agronomy, Iowa State University, Ames, Iowa, United States of America; Huazhong University of Science and Technology, CHINA

## Abstract

Kernel abortion is common phenomenon *in vivo* haploid induction and closely linked with haploid induction rate, but little information of kernel abortion is available and its genetic basis still unclear. We used two mapping populations including 186 and 263 F_2.3_ family lines to analyze the different degree of kernel abortion and identify quantitative trait loci (QTL) responsible for kernel abortion during haploid induction. In total 62 putative QTL, accounting for 3.27–14.70% of the phenotypic variation in kernel abortion traits, were detected across all 10 chromosomes. Ten QTL with over 10% contribution to phenotypic variation were affecting the fifth level of endosperm abortion (EnA5^th^), endosperm abortion (EnA) and total abortion (TA). Co-localization among kernel abortion traits QTL was observed in both populations and among different kernel abortion types. Five overlaps were indentified in the QTL for kernel abortion traits and HIR traits. Maize chromosome bins 3.01–3.02, 3.04–3.06, 4.05–4.06, 5.03–5.04, 8.06 were QTL hotspots for three or four traits related to the kernel abortion during haploid induction. Total kernel abortion rate (TAR) and HIR showed highly significant positive correlation. These findings may help to reveal haploid induction mechanisms and improve haploid production efficiency.

## Introduction

Defective kernels are a concern to breeders as kernel abortion reduces grain yield potential. Defective kernels can be caused either by physiological or reproductive factors. The former results from discordant flow of organic matter from source to sink. These could be source limits caused by insufficient sunlight or interrupted flow of dry matter accumulation and transportion caused by a disrupted vascular system in case of lodging. If this happens early 4–14 days after double fertilization, grain filling has not started. Thus there is no or little starch accumulated in the endosperm, resulting in formation of membranoid substance or yellow granule, which is called abortion tablet. If flow interruption happens15-25 days after double fertilization, grain filling starts but stops halfway, leading to limited starch accumulation in the endosperm, and shrunken grains, causing a diapause tablet [1]. In order to determine, how these kernel defects happen, several mutants were studied and causative genes cloned, one example is *defective kernel1* (*dek1*), required for aleurone cell development in the endosperm of maize grains, which encodes a membrane protein of the calpain gene superfamily [2,3,4]. Another example is *empty pericarp*4 (*emp4*), encoding a mitochondrion-targeted pentatricopeptide repeat protein necessary for seed development and plant growth in maize [5].

Reproductive abortion happens during fertilization. The *ig1* mutant produces defective kernels, when used as female parent [6,7]. Lin [8] suggested that this kind of kernel defect is caused by four or more excess polar nuclei existing in the embryo sac. This type of kernel abortion directly relates to double fertilization, which simultaneously initiates two major compartments of maize kernels, the embryo and the endosperm.

Doubled haploids are considered to be an effective way to accelerate maize breeding. Significant efforts were attributed to understand the genetic control of double fertilization, among others to enhance the rate of haploid production. Various QTL were detected in different populations [9,10]. The major gene responsible for haploid induction rate has been cloned by three independent groups and has been named matrilineal (MTL), ZmPHOSPHOLIPASE, A1, and NOT LIKE DAD (NLD), respectively [11,12,13]. Up to now, there are two ways both contributed to in vivo induced haploid [1]. Single fertilization as one possible mechanism [14,15] is defined as one of the two sperm cells failing to fuse with an egg cell but instead triggering haploid embryogenesis. An alternative mechanism is chromosome elimination [16]. In this case chromosomes from the inducer degenerate and are eliminated stepwise in primordial cells during subsequent cell divisions, after fusion of a sperm cell with an egg cell. Occurence of chromosome elimination has been confirmed [17,18,19]. In addition, inducibility (donor response to induction) have been shown to highly affect haploid induction [20,21]. Further aberrant reproductive and development related phenomena occur in the process of haploid induction, such as twin embryos [22,23,24]. Therefore, dedicated materials for haploid induction such as inducer lines originating from Stock 6 are used for research on double fertilization. However, haploid seed production is affected not only by the haploid induction rate, but also by several other factors. Defective kernels often accompany *in vivo* haploid induction [25]. Endosperm defective rate (EDR) affects HIR and the *sed1* (*segregation distortion1*) locus affecting endosperm abortion, co-segregates with *qhir1*. It was speculated that many of the aborted kernels could be haploid and that a high EDR reduces the haploid production [26]. However, little information is available for the genetic basis of kernel abortion by in vivo haploid induction. HIR was determined in two sets of F_2:3_ populations induced by two different inducer lines CAU5 and YHI-1, in order to study this problem from the maternal perspective (induced materials). The objectives of this study were to (1) detect the genetic basis of kernel abortion associated with haploid induction, (2) divide types of endosperm abortion caused by *in vivo* haploid induction into five different levels, (3) confirm whether kernel abortion is related to haploid induction.

## Materials and methods

### Plant materials and field experiments

Two sets of connected experimental materials were used in this study. For the first set of materials, maize haploid inducer line CAU5 [26] was used as male parent. The HIR of CAU5 is ~10%. The maternal donors were 186 F_2:3_ families obtained from the hybrid Zhengdan958 (ZD958) selfing, which was developed at Henan Academy of Agricultural Sciences, China. The parental lines are Zheng58 and Chang7-2. Induction crosses were performed manually at the Shangzhuang Experimental Station in Beijing (40°08′ N Lat., E116°10′ E Long), China in 2010. The second population includes 263 F_2:3_ families lines derived from the cross between Zheng58 and K22. Inbred line K22 was developed at Northwest Agriculture and Forestry University, China. Each family was crossed to inducer YHI-1 (Yu High Inducer No.1). YHI-1 has a HIR over 10%, and was developed at Henan Agricultural University. Crosses were carried out at Hainan experimental station of Henan Agricultural University (18°21N, 109°10E) during winter of 2013 and in Zhengzhou (34°80' N, 113°42' E), during summer of 2014, respectively. A randomized complete block design was utilized with two replications per genotype in two sets of populations. In each block, plants were sown in single rows with 3 m long and a 0.60 m distance between rows with 15 plants were included per row. Standard agronomic practices such as irrigation, fertilization, and weeding were used during the entire growth period.

### Kernel abortion phenotyping

**Fig 1:**
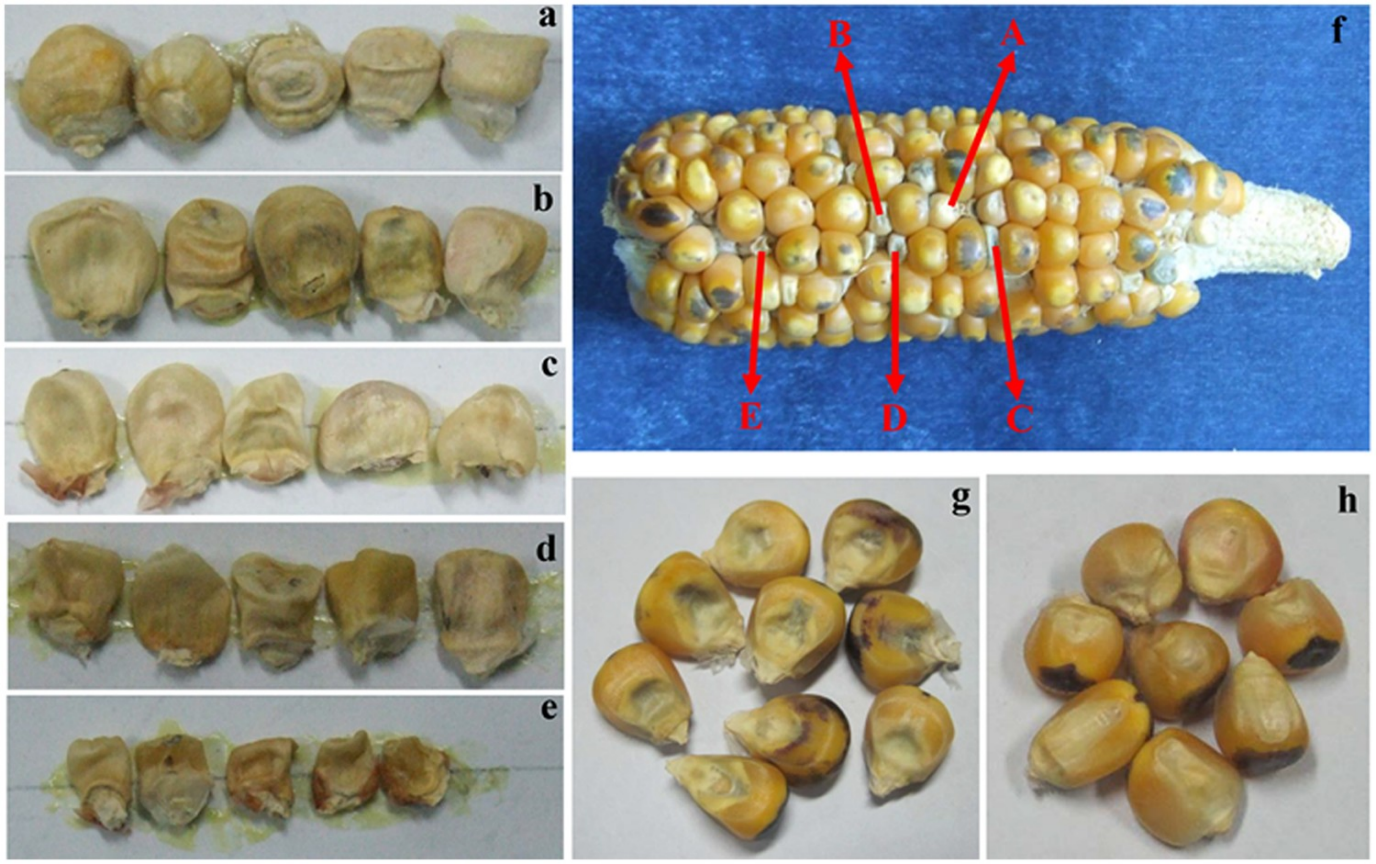
The embryo abortion and different degree of endosperm abortion caused by *in vivo* haploid induction. (a)1^st^ endosperm aborted kernels (EnA1^st^), same of the kernel A in Fig 1(f); (b) 2^nd^ endosperm aborted kernels (EnA2^nd^), same of the kernel B in Fig 1(f); (c) 3^rd^ endosperm aborted kernels (EnA3^rd^), same of kernel C in Fig 1(f); (d) 4^th^ endosperm aborted kernels (EnA4^th^), same of kernel D in Fig 1(f); (e) 5^th^ endosperm aborted kernels(EnA5^th^), same of kernel E in Fig 1(f); (f) F_1_ ear coming from one plant of Zhengdan958 F_2:3_ population crossing with inducer CAU5; (g) embryo aborted kernels; (h) haploid kernels.

A certain degree of kernel abortion is usually accompanied by haploid induction. There are two types of kernel abortion, one is embryo abortion (EmA) and the other is endosperm abortion (EnA). All kernels were assigned to one out of five levels of endosperm abortion (Fig 1): the first level (the highest level, EnA 1^st^), with the plumpness degree of grain reaching more than 80% of the normal kernel; the second level (EnA 2^nd^), with the plumpness degree of grain being 60%~80% of the normal kernel; the third level (EnA 3^rd^), with the plumpness degree being about 30%~60% of the normal kernel; the fourth level (EnA 4^th^), with the plumpness degree only accounting for 10%~30% of the normal kernel; the fifth and least level (EnA 5^th^): the plumpness degree was less than 10% of the normal kernel.

The proportion of these different level of aborted kernels were calculated as follows:

*EmAR%* = EmA kernels/total kernels (total kernels = total normal kernels + total embryo aborted kernels + total endosperm aborted kernels)× 100%; *EnA1*^*st*^*R%* = EnA 1^st^ kernels/total kernels× 100%; *EnA2*^*nd*^*R%* = EnA 2^nd^ kernels/total kernels × 100%; *EnA3*^*rd*^*R%* = EnA 3^rd^ kernels/total kernels × 100%; *EnA4*^*th*^*R%* = EnA 4^th^ kernels/total kernels × 100%; *EnA5*^*th*^*R%* = EnA 5^th^ kernels/total kernels × 100%; *Total EnAR%* (TEnAR%) = Total Endosperm aborted kernels/total kernels × 100%; Total kernel abortion rate (TAR%) = Total aborted kernels/total kernels × 100%.

The following mixed model was used for phenotypic data analysis:

$$Y_{ijk}=\mu +G_{i}+E_{j}+GE_{ij}+R{\left(E\right)}_{jk}+\varepsilon_{ijk}$$

Where, *Y*_*ijk*_ was the value of *i*th genotype under the *j*th environment and *k*th replication, *μ* was the overall population mean, *G*_*i*_ was the effect of genotype, *E*_*j*_ was the effect of the environment level, *GE*_*ij*_ was the effect of genotype by environment, *R*(*E*)_*jk*_ the effect of the *k*th replication in the *j*th enviroment, and ε_*ijk*_ was the error term. The heritability (*h*^2^) was estimated following by Hallauer and Miranda [27]:

$$h^{2}=\sigma^{2}{}_{G}/(\sigma^{2}{}_{G}+\sigma^{2}{}_{GE}/\text{j}+\sigma^{2}{}_{\varepsilon }/\text{jk})$$

Where σ^2^_*G*_ was the estimate of genotypic variance; σ^2^_*GE*_ the estimate of genotype × environment interaction variance; σ^2^_*ε*_ the estimate of error variance; j and k were the number of environments and replications in each environment, respectively.

### Molecular data collection and linkage map construction

Young leaves from F_2_ plants were obtained, flash-frozen in liquid N_2_, ground to a powder, and stored at -20 °C in individually labeled vials. Genomic DNA was extracted using a CTAB-based method described by Hoisington et al [28]. Simple sequence repeat (SSR) marker analysis was conducted as reported by Senior and Heun [29] using publicly available primers from the MaizeGDB (http://www.maizegdb.org). In our study, 130 and 158 polymorphic SSR markers with the coverage of the maize genome were selected respectively in both populations and used in the F_2_ population to develop a genetic linkage map, which was constructed with MAPMAKER 3.0 [30]. Recombination frequencies were converted into centi Morgans using the Kosambi mapping function [31].

### QTL analysis

WinQTL Cartographer V2.5 [32] was used to detect QTLs. The software uses the composite interval mapping (CIM) method [33]. The genome was scanned in 2 cM intervals using regression analysis. Default values of 5 for the control markers and 10 for the window size were used. A significance threshold for declaring a putative QTL was obtained from 1,000 permutations at *P* = 0.05 for each data set.

The QTL notation followed the rules suggested by McCouch *et al* [34]. Each QTL name was started with a lowercase ‘*q*’, then the trait name in capital letters, followed by a figure showing the chromosome number where the QTL was detected. If there were more than one QTL for the same trait on the same chromosome, a lowercase letter was added after the chromosome number to distinguish these QTL. In order to differentiate the QTL detected in different populations and environments, "*P1*" and "*P2*" appearing on the left bottom of QTL denoted as the QTL from the first and second population, respectively. "*HN*" and "ZZ" appearing on the left bottom of QTL denoted respectively as the QTL from the Hainan and Zhengzhou locations in the second population.

## Results

### Phenotypic data analysis

The total aborted kernel rate (TAR) for the male and female parents were quite different in each population. The same parent line Zheng58 was the high parent for TAR with 28.8% and 23.4% in population 1 and population 2, respectively. The inbred lines Chang7-2 and K22 were the low TAR parents, with 8.3% and 12.2%, respectively. In parent lines, except for EnA5^th^R, TEnAR and TAR, the other traits were below 4%. The average of TAR, TEnAR, EnA5^th^R, and EnA1^st^R of population 1 were 23.5, 17.8, 13.7 and 2.35%, and intermediate between both parents. Similar results were found in population 2, except for EnA1^st^R. In both populations, EnA3^rd^R values were lower than those of the low parent lines, while EmAR values were higher than the high parent (Table 1).

**Table 1:** Estimation of statistical parameters for different abortion traits.

Population	Traits	P1	P2	Family lines					
		Average	Average	Average	Max	Min	Range	S.D.	C.V.
Population 1	EnA1^st^ R	2.65	0.64	2.35	11.08	0.22	10.86	1.35	57.45
	EnA2^nd^R	1.33	0.92	0.49	4.90	0.00	4.90	0.59	122.04
	EnA3^rd^R	0.88	0.64	0.27	5.85	0.00	5.85	0.51	189.51
	EnA4^th^R	2.21	1.10	0.95	18.82	0.00	18.82	1.61	168.70
	EnA5 ^th^R	20.35	4.70	13.71	46.50	2.54	43.96	7.37	53.78
	TEnAR	27.43	8.01	17.77	52.09	4.98	47.11	8.22	46.27
	EmAR	1.33	0.28	5.69	14.66	2.24	12.42	1.62	28.51
	TAR	28.76	8.29	23.46	60.27	9.10	51.17	8.12	34.63
Population 2	EnA1^st^R	1.02	0.94	0.86	3.88	0.00	3.88	0.63	72.98
	EnA2^nd^R	0.56	0.31	0.66	2.70	0.00	2.70	0.55	82.71
	EnA3^rd^R	1.58	1.36	0.95	5.15	0.00	5.15	0.71	75.04
	EnA4^th^R	1.09	0.80	1.19	8.55	0.00	8.55	1.29	108.32
	EnA5 ^th^R	16.18	5.18	6.93	24.41	0.00	24.41	3.87	55.83
	TEnAR	20.43	8.59	10.58	27.52	2.23	25.29	4.48	42.40
	EmAR	3.56	3.01	4.62	17.58	1.54	16.04	2.29	49.58
	TAR	23.43	12.16	15.20	33.48	5.55	27.93	5.02	33.03

EnA1^st^R, rate of the first level for endosperm abortion; EnA2^nd^R, rate of the second level for endosperm abortion; EnA3^rd^R, rate of the third level for endosperm abortion; EnA4^th^R, rate of the fourth level for endosperm abortion; EnA5^th^R, rate of the fifth level for endosperm abortion; TEnAR, rate of total endosperm abortion; EmAR, rate of embryo abortion; TAR, rate of total abortion. The same below. P1, the parent line Zheng58 in both population, P2, Chang7-2 and K22 in the first and second population, respectively. SD, standard deviation; CV, coefficient of variance.

All variance components for genotype, environment and G × E interactions were highly significant for all kernel abortion traits (Table 2). The heritability for EnA1^st^R, EnA2^nd^R, and EnA3^rd^R were 73.5, 64.6, and 58.6%, respectively. Those of other traits were about 50% (Table 2). This suggests that kernel abortion by induction is not only affected by genetic background but also by environment.

**Table 2:** Anova of different types of abortion kernels in both populations.

Compoents	EnA1^st^R	EnA2^nd^R	EnA3^rd^R	EnA4^th^R	EnA5 ^th^R	TEnAR	EmAR	TAR
Genotype	0.67**	0.28**	0.3**	0.7**	9.01**	12.1**	2.74**	14.31**
Environment	0.83**	0.01**	0.16**	0.07**	18.71**	22.33**	1.61**	33.88**
Genotype × Environmet	0.22**	0.16**	0.28**	0.87**	14.84**	19.52**	5.69**	27.81**
Error	1.01**	0.60**	0.71**	2.15**	18.70**	22.43**	6.23**	25.69**
Heritability	73.49	64.62	58.63	51.92	52.77	54.15	48.28	51.36

** reached significant at 0.01 level.

### Correlations among different types of kernels

TAR had a significantly positive correlation with every different level (from EnA1^st^ to EnA5^th^) of defective kernels except for EmAR of the first population and EnA1^st^R of the second population. The correlation coefficients between TAR and TEnAR were high (0.98 and 0.89) in both populations, respectively. TEnAR had a significant positive correlation with every different level (from 1^st^ to 5^th^) except for EnA1^st^R in population 2. The correlation coefficient between TEnAR and EnA5^th^R was 0.95 and 0.92 in populations 1 and 2 (Table 3), respectively. This suggests that during *in vivo* haploid induction, defective kernels occurred mostly from endosperm abortion, and aborted endosperm mostly depends on the 5^th^ level of endosperm aborted kernels. Correlation analysis between defective kernel traits and haploid inducibility showed that TAR had a significant positive correlation with haploid induction rate (HIR) and coefficients of correlation reached 0.35 and 0.16 in both populations, respectively. Significant positive correlations were found between HIR, TEnAR, and EnA3^rd^R in population 1. A highly significant positive correlation was also found between EmAR and HIR in population 2 (Table 3).

**Table 3:** Correlations between different types of abortion kernels.

Trait	EnA1^st^R	EnA2^nd^R	EnA3^rd^R	EnA4^th^R	EnA5 ^th^R	EmAR	TEnAR	TAR	HIR
EnA1^st^R	1	0.12	0.13*	0.16**	0.08	0.16*	0.01	0.06	0.05
EnA2^nd^R	0.30**	1	0.19**	0.17**	-0.02	0.14*	0.17**	0.21**	0.00
EnA3^rd^R	0.05	0.06	1	0.34**	0.06	0.07	0.32**	0.31**	-0.06
EnA4^th^R	0.09	0.19*	0.17*	1	0.21**	0.04	0.51**	0.47**	0.03
EnA5 ^th^R	0.06	-0.02	0.09	0.20**	1	-0.07	0.92**	0.78**	0.09
EmAR	0.14*	0.00	0.05	-0.06	-0.19**	1	-0.01	0.45**	0.18**
TEnAR	0.26**	0.15*	0.19*	0.42**	0.95**	0.16*	1	0.89**	0.09
TAR	0.29**	0.15*	0.20**	0.41**	0.92**	0.04	0.98**	1	0.16**
HIR	0.02	-0.06	0.17*	0.04	0.00	-0.03	0.34**	0.35**	1

The lower left part for the first population and the upper right part for the second population, HIR, haploid induction rate.

### Identified QTL

**Fig 2:**
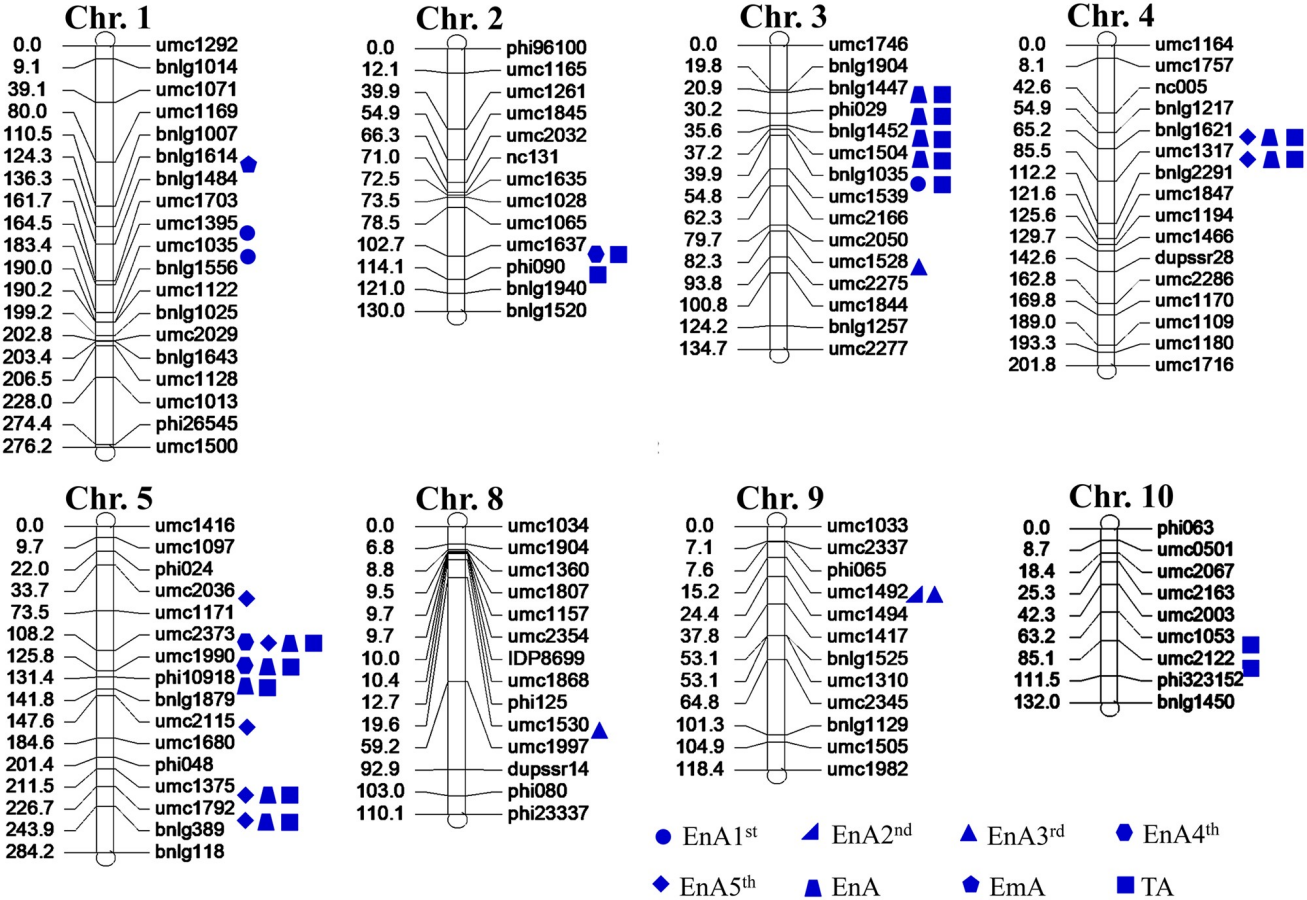
Distribution of QTL for kernel abortion related during parthenogenesis induced process in the first population (Zheng58×Chang7-2). The circle means QTLs for the 1^st^ endosperm abortion kernels (EnA1^st^); the right triangle means QTLs for the 2^nd^ endosperm aborted kernels (EnA2^nd^), the equilateral triangle means QTLs for the 3^rd^ Endosperm aborted kernels (EnA3^rd^), the hexagon means QTLs for the 4^th^ endosperm aborted kernels (EnA4^th^), The rhombus means QTLs for the 5^th^ endosperm aborted kernels (EnA5^th^), the trapezoid means QTLs for the total endosperm aborted kernels (EnA). The pentagon means QTLs for the embryo abortion kernels (EmA). The square means QTLs for the total aborted kernels (TA).

**Fig 3:**
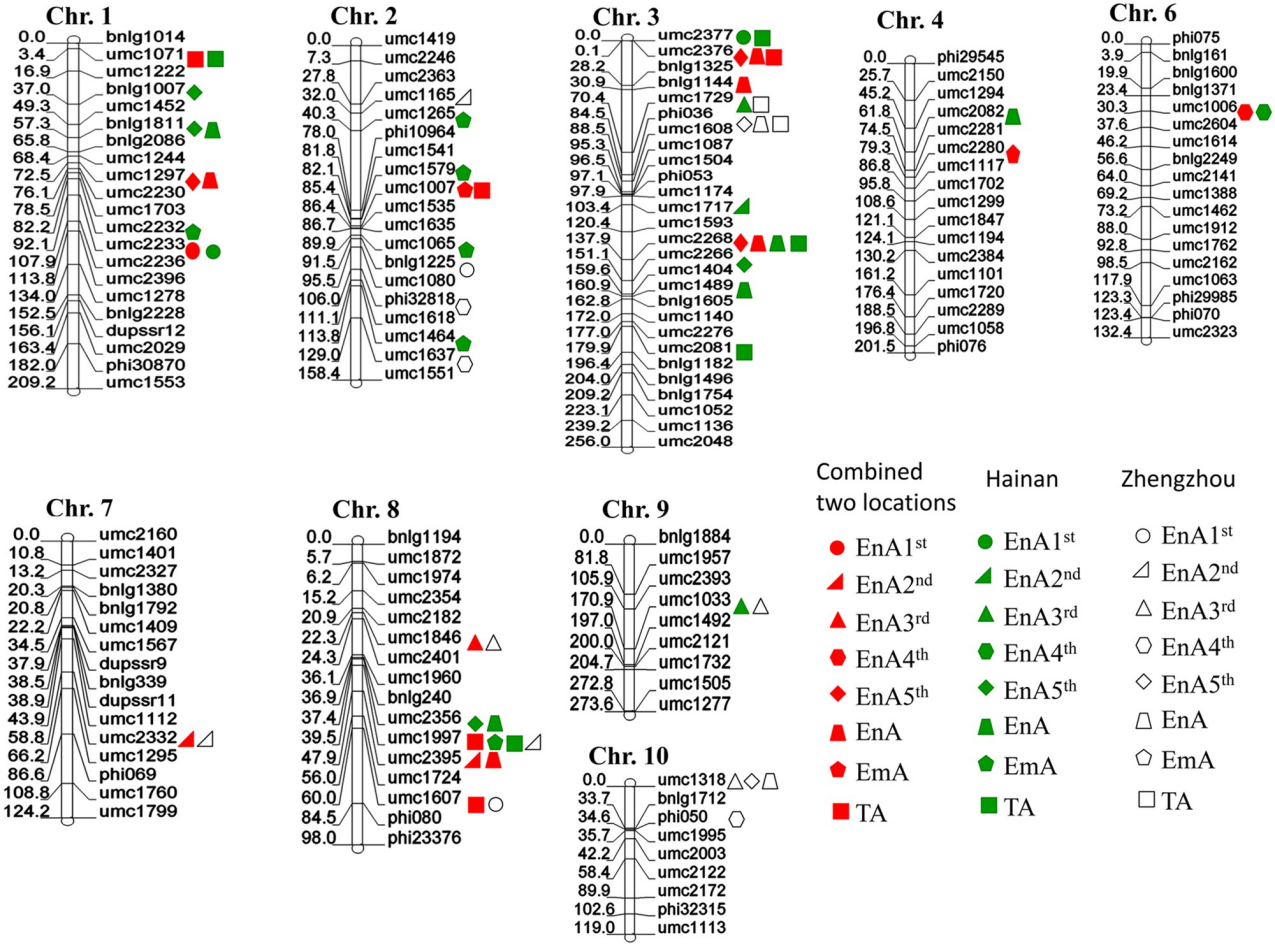
Distribution of QTL for kernel abortion related during parthenogenesis induced process in the second population (Zheng58×K22). The circle means QTLs for the 1^st^ endosperm abortion kernels (EnA1^st^); the right triangle means QTLs for the 2^nd^ endosperm aborted kernels (EnA2^nd^), the equilateral triangle means QTLs for the 3^rd^ Endosperm aborted kernels (EnA3^rd^), the hexagon means QTLs for the 4^th^ endosperm aborted kernels (EnA4^th^), The rhombus means QTLs for the 5^th^ endosperm aborted kernels (EnA5^th^), the trapezoid means QTLs for the total endosperm aborted kernels (EnA). The pentagon means QTLs for the embryo abortion kernels (EmA). The square means QTLs for the total aborted kernels (TA). The red means the QTL identified across two environments, the green means the QTL identified in Hainan environment; the white means the QTL identified in Zhengzhou environment.

For population 1,528 SSR markers from across the maize genome were screened for the two parental lines, Zheng58 and Chang7-2, 130 polymorphic SSR markers were selected and used to develop the genetic map. The map covered a total length of 1,553 centi morgan (cM), with an average distance of 12.0 cM between markers (Fig 2). For population 2, 1200 SSR markers from across the maize genome were screened 158 of these SSR markers were polymorphic between the two parental lines Zheng58 and K22, and were used to develop the genetic map. The map covered a total length of 1,649 cM, with an average distance of 10.4 cM between markers (Fig 3). These two linkage maps were used to identify QTL.

A total 62 QTL related to kernel abortion were detected in both populations, 42 QTL in population 1 and 20 QTL in population 2. These QTL were distributed over 10 chromosomes. The number of QTL on chromosomes 3 and 5 was highest: 17 and 15 QTL were identified on each chromosome, there was only one QTL detected on each of chromosomes 6 and 7, and two QTL on each of chromosomes 9 and 10. 16 and 5 QTL for TA were detected in population 1 and population 2, respectively. These in total sixteen QTL were distributed over chromosomes 1, 2, 3, 4, 5, 8, 10. Six, five and three QTL were identified on chromosomes 3, 5 and 2, respectively. There were two QTL on each of chromosomes 4, 8, and 10. Only one QTL was detected on chromosome 1. Fourteen of the QTL, showed partial dominance, five of them showed additive effects, the remaining two displayed over-dominance. The QTL for TA between SSR markers umc2373 and umc1990 on chromosome 5 in population 1 shared the same region with three QTL for EnA4^th^, EnA5^th^ and EnA. The QTL for TA between SSR markers umc2376 and bnlg1325 on chromosome 3 in the second population shared the same region with the other two QTL for EnA5^th^ and EnA. Sixteen QTL for the EnA were identified, seven and five of them on chromosomes 3 and 5, respectively. There were two QTL on chromosome 4. Only one QTL was detected on each of chromosomes 1 and 8. The percentage of contribution of all these QTL for TA and EnA to the phenotypic variation was below 10% except for two QTL on chromosomes 4 between bnlg1621 and umc1317, umc1317 and bnlg2291, and one QTL between umc2373 and umc1990 on chromosome 5, shared by both traits. Three QTL were detected for EmA on chromosomes 1, 2, 4. The QTL were flanked by SSR markers umc1007 and umc1535 on chromosome 2 shared the same region with the QTL for TA, but with lower contribution to phenotypic variation (Table 4). For different levels of endosperm abortion (1^st^- 5^th^), the number of QTL for En5^th^ (10) was higher than for others, four QTL for EnA1^st^ and EnA4^th^, three QTL for EnA2^nd^ and EnA 3^rd^ were identified. Most of these QTL not share the same region except for the QTL for EnA2^nd^ and EnA3^rd^ sharing the region flanked by markers umc1492-umc1494 and QTL for EnA4^th^ and EnA5^th^ shared the region flanked by markers umc2373-umc1990. The contributions of only four QTL for EnA5^th^ exceeded 10%. These QTL were located on chromosomes 4 and 5, with two on each chromosome, respectively, three with additive gene action, the other one with partial dominance.

**Table 4:** QTL Identified for different abortion of endosperm and embryo.

Trait	QTL	Flanking markers	Position	LOD	A	D	R^2^	|D|/|A|	Gene Action
EnA1^st^	_*P1*_*qEnA1*^*st*^*-1a*	umc1395-umc1035	181.51	3.38	0.71	-0.45	7.51	0.63	PD
	_*P1*_*qEnA1*^*st*^*-1b*	umc1035-bnlg1556	183.41	3.35	0.68	-0.44	6.93	0.65	PD
	_*P2*_*qEnA1*^*st*^*-1*	umc2233-umc2236	104.11	2.88	-0.06	-0.20	5.55	3.33	OD
	_*P1*_*qEnA1*^*st*^*-3*	bnlg1035-umc1539	42.91	2.70	0.68	-0.37	6.94	0.54	PD
EnA2^nd^	_*P2*_*qEnA2*^*nd*^*-7*	umc2332-umc1295	63.81	4.10	-0.07	-0.2	7.43	2.86	OD
	_*P2*_*qEnA2*^*nd*^*-8*	umc2395-umc1724	47.91	4.67	0.21	-0.03	7.18	0.14	A
	_*P1*_*qEnA2*^*nd*^*-9*	umc1492-umc1494	15.21	2.52	-0.15	0.32	4.70	2.13	OD
EnA3^rd^	_*P1*_*qEnA3*^*rd*^*-3*	umc1528-umc2275	82.31	2.56	-0.06	0.18	5.52	3.00	OD
	_*P2*_*qEnA3*^*rd*^*-8*	umc1846-umc2401	22.31	3.07	-0.01	0.24	4.89	17.14	OD
	_*P1*_*qEnA3*^*rd*^*-9*	umc1492-umc1494	15.21	2.52	-0.09	0.18	5.67	2.00	OD
EnA4^th^	_*P1*_*qEnA4*^*th*^*-2*	umc1637-phi090	108.71	2.54	0.55	-0.42	6.45	0.76	PD
	_*P1*_*qEnA4*^*th*^*-5a*	umc2373-umc1990	122.21	3.88	-0.33	-0.29	9.59	0.88	D
	_*P1*_*qEnA4*^*th*^*-5b*	umc1990-phi109188	125.81	3.76	-0.34	-0.25	8.19	0.74	PD
	_*P2*_*qEnA4*^*th*^*-6*	umc1006-umc2604	30.31	2.54	0.08	0.35	3.27	4.38	OD
EnA5^th^	_*P2*_*qEnA5*^*th*^*-1*	umc1297-umc2230	72.51	6.18	2.27	-1.44	9.07	0.63	PD
	_*P2*_*qEnA5*^*th*^*-3a*	umc2268-umc2266	149.91	4.75	-1.33	-0.6	7.42	0.45	PD
	_*P2*_*qEnA5*^*th*^*-3b*	umc2376-bnlg1325	1.06	3.85	1.13	-1.99	5.8	1.76	OD
	_*P1*_*qEnA5*^*th*^*-4a*	bnlg1621-umc1317	74.21	5.33	3.26	0.32	12.37	0.10	A
	_*P1*_*qEnA5*^*th*^*-4b*	umc1317-bnlg2291	99.51	5.49	4.01	-0.68	14.7	0.17	A
	_*P1*_*qEnA5*^*th*^*-5a*	umc2373-umc1990	121.21	3.00	-1.58	-1.77	8.14	1.12	D
	_*P1*_*qEnA5*^*th*^*-5b*	umc1375-umc1792	219.51	6.06	-3.67	0.21	14.10	0.06	A
	_*P1*_*qEnA5*^*th*^*-5c*	umc1792-bnlg389	229.71	5.64	-3.70	1.01	12.45	0.27	PD
EnA	_*P2*_*qEnA-1*	umc1297-umc2230	72.51	6.00	2.38	-1.23	8.79	0.52	PD
	_*P1*_*qEnA-3a*	bnlg1447-phi029	29.91	3.79	3.52	-0.78	6.80	0.22	PD
	_*P1*_*qEnA-3b*	phi029-bnlg1452	31.21	3.98	3.78	-0.86	7.70	0.23	PD
	_*P1*_*qEnA-3c*	bnlg1452-umc1504	36.61	3.88	3.63	-0.93	7.26	0.26	PD
	_*P1*_*qEnA-3d*	umc1504-bnlg1035	37.21	4.16	3.56	-1.02	7.32	0.29	PD
	_*P2*_*qEnA-3a*	umc2268-umc2266	148.91	4.51	-1.78	-0.35	7.36	0.20	A
	_*P2*_*qEnA-3b*	bnlg1144-umc1729	67.91	3.00	2.15	-0.78	5.75	0.36	PD
	_*P2*_*qEnA-3c*	umc2376-bnlg1325	4.06	2.59	1.15	-2.01	4.73	1.75	OD
	_*P1*_*qEnA-4a*	bnlg1621-umc1317	80.21	5.42	3.58	0.45	11.20	0.13	A
	_*P1*_*qEnA-4b*	umc1317-bnlg2291	91.51	5.36	3.77	0.17	11.58	0.05	A
	_*P1*_*qEnA-5a*	umc2373-umc1990	120.21	5.76	-2.29	-2.68	14.42	1.17	D
	_*P1*_*qEnA-5b*	umc1990-phi109188	125.81	4.71	-2.40	-1.51	8.17	0.63	PD
	_*P1*_*qEnA-5c*	phi109188-bnlg1879	135.41	3.39	-2.77	-0.47	6.72	0.17	A
	_*P1*_*qEnA-5d*	umc1375-umc1792	224.51	4.61	-3.08	-0.43	8.82	0.14	A
	_*P1*_*qEnA-5e*	umc1792-bnlg389	228.71	4.65	-3.10	-0.26	8.90	0.08	A
	_*P2*_*qEnA-8*	umc2395-umc1724	47.91	4.81	1.99	-1.16	7.02	0.58	PD
EmA	_*P1*_*qEmA-1*	bnlg1614-bnlg1484	124.31	2.56	1.03	-0.31	4.08	0.30	PD
	_*P2*_*qEmA-2*	umc1007-umc1535	85.41	2.71	-0.04	-0.81	4.2	20.25	OD
	_*P2*_*qEmA-4*	umc2280-umc1117	85.31	3.35	0.98	-0.53	6.14	0.54	PD
TA	_*P2*_*qTA-1*	umc1071-umc1222	15.41	3.05	2.00	-0.59	5.08	0.30	PD
	_*P1*_*qTA-2a*	umc1637-phi090	1137.1	2.65	2.57	-0.46	4.49	0.18	A
	_*P1*_*qTA-2b*	phi090-bnlg1940	116.11	2.74	2.70	-0.62	4.87	0.23	PD
	_*P2*_*qTA-2*	umc1007-umc1535	85.41	2.79	-0.83	-1.24	4.12	1.49	OD
	_*P1*_*qTA-3a*	bnlg1447-phi029	29.91	2.88	3.28	-1.23	5.01	0.38	PD
	_*P1*_*qTA-3b*	phi029-bnlg1452	31.21	3.00	3.44	-1.22	5.58	0.35	PD
	_*P1*_*qTA-3c*	bnlg1452-umc1504	36.61	2.78	3.11	-0.85	5.01	0.27	PD
	_*P1*_*qTA-3d*	umc1504-bnlg1035	39.21	3.40	3.19	-0.81	5.99	0.25	PD
	_*P1*_*qTA-3e*	bnlg1035-umc1539	39.91	3.37	3.06	-0.74	5.70	0.24	PD
	_*P2*_*qTA-3*	umc2376-bnlg1325	3.06	3.42	1.27	-2.58	6.03	2.03	OD
	_*P1*_*qTA-4a*	bnlg1621-umc1317	84.21	5.71	3.03	0.84	10.24	0.28	PD
	_*P1*_*qTA-4b*	umc1317-bnlg2291	86.51	5.70	3.03	0.78	10.19	0.26	PD
	_*P1*_*qTA-5a*	umc2373-umc1990	121.21	4.85	-2.89	-1.51	10.82	0.52	PD
	_*P1*_*qTA-5b*	umc1990-phi109188	127.81	4.65	-3.01	-0.85	8.50	0.28	PD
	_*P1*_*qTA-5c*	phi109188-bnlg1879	136.41	4.19	-3.23	-0.20	8.09	0.06	A
	_*P1*_*qTA-5d*	umc1375-umc1792	223.51	3.37	-2.84	-0.04	6.37	0.01	A
	_*P1*_*qTA-5e*	umc1792-bnlg389	231.71	3.52	-3.02	0.30	6.87	0.10	A
	_*P2*_*qTA-8a*	umc1997-umc2395	45.51	6.00	2.48	-0.33	8.5	0.13	A
	_*P2*_*qTA-8b*	umc1607-phi080	71.01	2.92	-2.27	0.47	7.06	0.21	PD
	_*P1*_*qTA-10a*	umc1053-umc2122	81.21	2.74	-3.09	1.81	5.23	0.59	PD
	_*P1*_*qTA-10b*	umc2122-phi323152	92.11	2.78	-3.31	2.19	5.86	0.66	PD

Gene action according to DR = |D|/|A|, additive(A), DR<0.2; partially dominant(PD)0.2≤DR<0.8; dominant(D), 0.8≤DR<1.2 over dominant(OD),1.2≤DR. P1, the QTL indentified in the first population; p2, the QTL indentified in the second population.

Populations evaluated in two locations have significantly environment differences. A total of 43 QTL were detected across both environments (Table 5). 17 and 26 QTL for all traits of kernel abortion were identified in Zhengzhou and Hainan, respectively. These QTL were distributed over nine chromosomes except for chromosome 5. Most of the QTL were located on chromosomes 1, 3, and 8, with 6, 13, and 7 QTL, respectively (Fig 3). Each QTL explained 3.6% to 17.0%, including two QTL with more than 15% contribution to the phenotype variation. There was no consistent QTL for any of the traits detected in both locations except for EnA3^rd^ on chromosome 9, but some co-localization of the abortion QTL were observed. Two regions of flanking markers umc2266-umc2268 on chromosome 3 and umc1997-umc2395 on chromosome 8 had four QTL detected, respectively. Three QTL with the same region flanked by markers umc2376-bnlg1325, phi036-umc1608 on chromosome 3 and umc1318-bnlg1712 on chromosome 10, were detected.

**Table 5:** QTL Identified for different abortion of endosperm and embryo of the second population in single environment.

Trait	QTL	Flanking markers	Position	LOD	A	D	R^2^	|D|/|A|	Gene Action
EnA1^st^	_*HN*_*qEnA1*^*st*^*-1*	umc2233-umc2236	107.11	2.74	-0.10	-0.22	4.54	2.20	OD
	_*ZZ*_*qEnA1*^*st*^*-2*	bnlg1225-umc1080	93.51	2.62	-0.17	-0.16	3.76	0.94	D
	_*HN*_*qEnA1*^*st*^*-3*	umc2377-umc2376	0.01	2.50	0.23	-0.08	3.96	0.35	PD
	_*ZZ*_*qEnA1*^*st*^*-8*	umc1607-phi080	67.01	2.52	-0.17	-0.20	4.87	1.18	D
EnA2^nd^	_*ZZ*_*qEnA2*^*nd*^*-2*	umc1165-umc1265	32.01	3.19	0.3	0.03	4.81	0.10	A
	_*HN*_*qEnA2*^*nd*^*-3*	umc1717-umc1593	88.21	3.83	-0.17	-0.11	6.32	0.65	PD
	_ZZ_qEnA2^nd^-7	umc2332-umc1295	63.81	3.08	-0.2	-0.19	5.66	0.95	D
	_ZZ_qEnA2^nd^-8	umc1997-umc2395	45.51	2.65	0.24	-0.07	4.51	0.29	PD
EnA3^rd^	_*HN*_*qEnA3*^*rd*^*-3*	umc1729-phi036	84.41	2.68	0.95	-0.20	4.41	0.21	PD
	_ZZ_qEnA3^rd^-8	umc1846-umc2401	22.31	2.58	0.18	0.16	3.59	0.89	D
	_*HN*_*qEnA3*^*rd*^*-9*	umc1033-umc1492	170.91	3.04	-0.12	-0.28	5.20	2.33	OD
	_ZZ_qEnA3^rd^-9	umc1033-umc1492	105.91	2.83	0.34	-0.05	16.99	0.15	A
	_ZZ_qEnA3^rd^-10	umc1318-bnlg1712	0.01	2.55	0.10	-0.24	3.55	2.40	OD
EnA4^th^	_ZZ_qEnA4^th^-2a	umc1637-umc1551	129.01	5.15	0.49	-0.32	8.48	0.65	PD
	_ZZ_qEnA4^th^-2b	phi32818-umc1618	106.01	2.51	-0.33	-0.27	3.95	0.82	D
	_*HN*_*qEnA4*^*th*^*-6*	umc1006-umc2604	35.31	2.87	0.21	0.71	4.94	3.38	OD
	_ZZ_qEnA4^th^-10	phi050-umc1995	34.61	2.56	-0.29	-0.10	3.76	0.34	PD
EnA5^th^	_*HN*_*qEnA5*^*th*^*-1a*	bnlg1811-bnlg2086	60.31	6.19	3.38	-2.11	9.48	0.62	PD
	_*HN*_*qEnA5*^*th*^*-1b*	bnlg1007-umc1452	49.01	4.60	2.84	-1.91	6.51	0.67	PD
	_*HN*_*qEnA5*^*th*^*-3*	umc2266-umc1404	151.11	4.50	-1.27	-1.00	6.03	0.79	PD
	_ZZ_qEnA5^th^-3	phi036-umc1608	87.51	2.88	0.58	-1.99	5.31	3.43	OD
	_*HN*_*qEnA5*^*th*^*-8*	umc2356-umc1997	37.41	3.92	2.03	-0.29	5.29	0.14	A
	_ZZ_qEnA*5*^*th*^-10	umc1318-bnlg1712	0.01	2.73	0.11	-1.98	4.70	18.00	OD
EnA	_*HN*_*qEnA-1*	bnlg1811-bnlg2086	59.31	3.77	3.07	-1.97	5.92	0.64	PD
	_*HN*_*qEnA-3a*	umc2268-umc2266	148.91	3.92	-0.98	-1.9	5.78	1.94	OD
	_HN_qEnA-3b	umc1489-bnlg1605	160.91	2.91	-1.03	-1.18	4.07	1.15	D
	_*ZZ*_*qEnA-3*	phi036-umc1608	87.51	3.31	1.02	-2.21	5.91	2.17	OD
	_HN_qEnA-4	umc2082-umc2281	61.81	2.83	-2.26	1.02	3.92	0.45	PD
	_HN_qEnA-8	umc2356-umc1997	37.41	3.50	2.09	-0.1	4.93	0.05	A
	_*ZZ*_*qEnA-10*	umc1318-bnlg1712	0.01	2.97	0.22	-2.30	5.03	10.45	OD
EmA	_*HN*_*qEmA-1*	umc2232-umc2233	83.21	2.81	-1.15	0.64	4.40	0.56	PD
	_*HN*_*qEmA-2a*	umc1065-bnlg1225	90.91	5.88	-1.26	-0.84	8.98	0.67	PD
	_*HN*_*qEmA-2b*	umc1579-umc1007	85.11	5.44	-0.88	-1.01	8.36	1.15	D
	_*HN*_*qEmA-2c*	umc1265-phi109642	77.31	4.74	-1.07	-0.77	7.48	0.72	PD
	_*HN*_*qEmA-2d*	umc1464-umc1637	128.81	3.04	1.31	-1.00	4.66	0.76	PD
	_*HN*_*qEmA-8*	umc1997-umc2395	39.51	2.60	0.98	-0.06	3.88	0.06	A
TA	_*HN*_*qTA-1*	umc1071-umc1222	12.41	2.84	2.97	-0.59	5.56	0.20	PD
	_*HN*_*qTA-3a*	umc2081-bnlg1182	186.91	6.96	-1.61	-2.74	15.44	1.70	OD
	_*HN*_*qTA-3b*	umc2268-umc2266	146.91	2.59	-0.17	-2.45	4.4	14.41	OD
	_*HN*_*qTA-3c*	umc2377-umc2376	0.01	2.58	1.36	-3.08	3.68	2.26	OD
	_*ZZ*_*qTA-3a*	umc1729-phi036	78.41	2.97	1.23	-2.66	5.87	2.16	OD
	_*ZZ*_*qTA-3b*	phi036-umc1608	87.51	3.57	1.43	-2.45	6.39	1.71	OD
	_*HN*_*qTA-8*	umc1997-umc2395	43.51	4.21	3.01	-0.35	7.23	0.12	A

Left the subscript "HN" and "ZZ" for each QTL represented two environments Hainan and Zhengzhou, respectively.

## Discussion

### Kernel abortion during in vivo haploid induction is under genetic control

Haploid production is hampered by defective kernels produced during in vivo haploid induction. Our results suggest that kernel abortion occurs during haploid induction is controlled by several QTL and is related to haploid induction. However, kernel abortion also depends on the maternal plants used as haploid donor germplasm. Defective kernels as a quantitative trait were detected respective QTL. Previously, defective kernel (endosperm or embryo) or kernel abortion was considered as a qualitative trait controlled by a single or few genes [35]. There are many defective kernel mutants and more than half of them have already been located on maize chromosomes. Some of them affect aleurone cell development [2], some alter the transfer cell layer gene expression in endosperm [36], others result in tiny amounts of floury starch [37]. All of them display morphological abnormalities in seed, and can even be lethal [38]. Although there are differences among these different kinds of kernel abortion, or even the differences present in one kind of defective trait, they are controlled by a limited number of qualitative trait genes and the differences are due to dosage effects. Here we analyzed kernel abortion as quantitative trait—kernel abortion rate (endosperm abortion rate and embryo abortion rate) developed during *in vivo* haploid induction. Endosperm abortion rate was divided into five degrees of abortion (from EnA1^st^R to EnA5^th^R), according to the different shape and amount of filling inside of kernels. In the present study, all of the mean kernel abortion rates (from EnA1^st^R to EnA5^th^R, TEnAR, EmAR and TAR) of Zheng58 were higher than those of Chang7-2 and K22. Especially the differences of mean values in EnA5^th^R, EnAR and TAR between both parents exceeded 10 percent. Except for some endosperm abortion traits (from EnA2^nd^R to En4^th^R in the Zheng58×Chang7-2 F_2:3_ population and EnA1^st^ and EnA3^rd^ in the Zheng58×K22 F_2:3_ population), the mean of kernel abortion traits for F_2:3_ families was higher than that of the low-parent. Moreover, individual F_2:3_ families transgressed their parents for all traits. The coefficient of variation for all of the traits exceeded 20%.

There were significant differences in different genotypes, environments and the interaction of genotype by environment for all kernel abortion traits. The effect of environments was the highest, followed by genotypes and the interaction between genotype and environment. The heritability of EnA1^st^ and EnA2^nd^ exceeded 60%. Thus, these abortion traits depend not only on genotype but also environmental effects. TEnAR, EmAR and TAR are closely correlated with each other. TAR was closely correlated with each degree of endosperm abortion, except for EnA1^st^R in the second population (r = 0.06) and EmAR in the first population (r = -0.03). EnA5^th^R was significantly correlated with EnA4^th^R, EnA4^th^R with EnA2^nd^R and EnA3^rd^R, and EnA1^st^R with EmA in both populations. This is consistent with results of QTL detection, some QTL of correlated traits shared the same genome regions (Figs 2 and 3).

### Relationship between kernel abortion and inducibility

**Fig 4:**
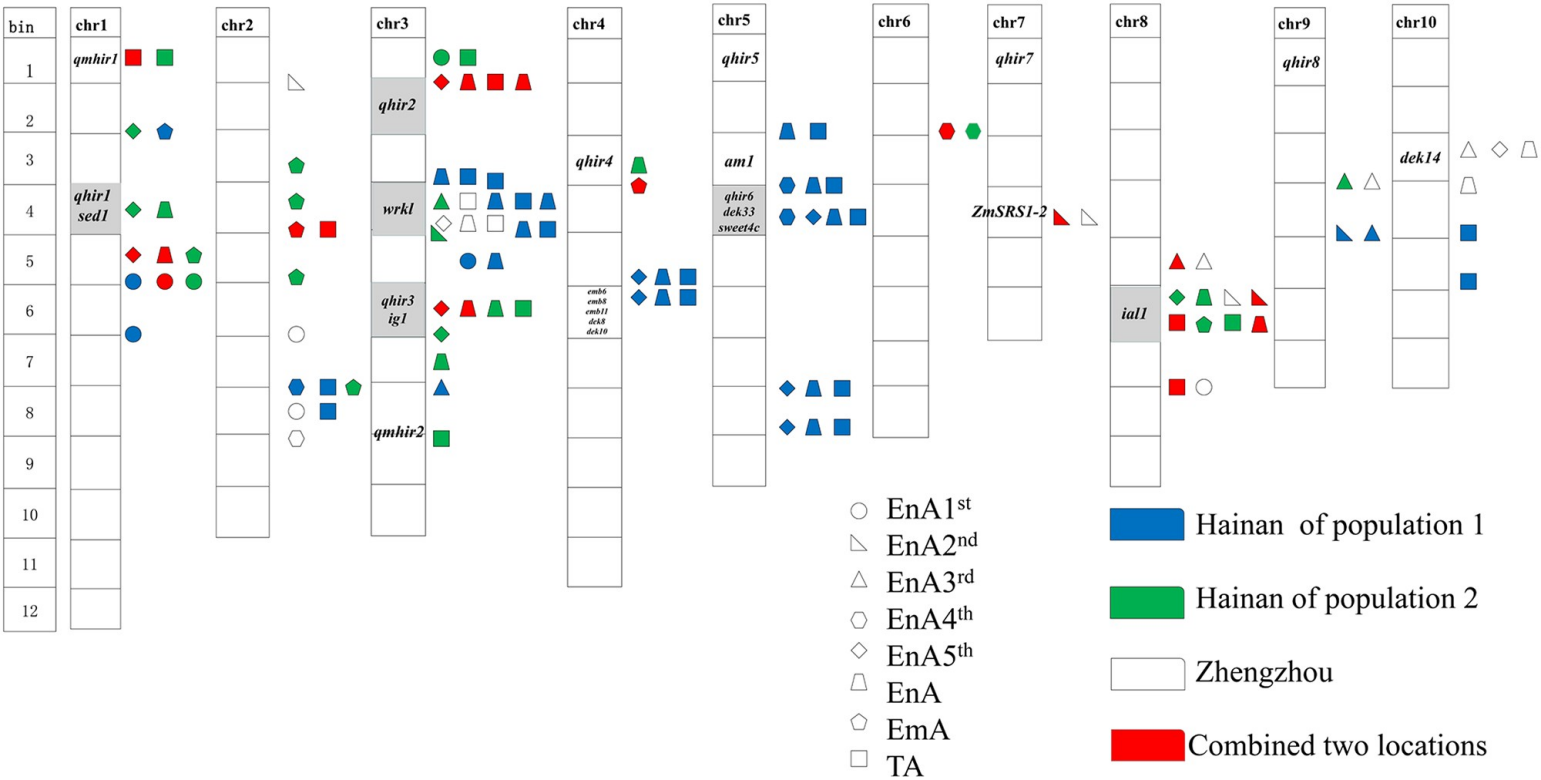
Distribution of QTL or genes for induction and kernel abortion. The yellow region stands for the genes or QTL related maize haploid production.

HIR and TAR are closely correlated (Table 3), which could be useful to predict the inducing ability of inducers, but may also complicate development and maintenance of inducers with higher HIR. Therefore, kernel abortion was also first emphasized as the genetic characteristic when studying haploid induction gene *qhir1* [26]. Both *qhir1* and *sed1* shared the same region on chromosome 1 and have a similar characteristics regarding phenotypic distribution, segregation distortion, when the donor material is induced by several inducers. It was proposed that *sed1* and *qhir1* may be the same pleiotropic gene. Furthermore, we compared QTL for kernel abortion caused by in vivo haploid induction with the reported QTL of maternal and paternal haploid induction rates (HIR) [9,10,20,39] according to physical position of the reference sequence for B73 (Fig 4). According to the current study, the paternal haploid induction rate were identified on chromosome 1, 3, 4, 5, 7 and 9 respectively, the most QTLs for kernel abortion were identified on chromosome 1, 3, 5 and 8. There were several QTL of kernel abortion from *in vivo* haploid induction overlapping or closely linked to QTL for HIR and maternal haploid induction rate (MHIR). For example, both of the *qhir1* and *sed1* shared the same region on chromosome 1 and overlapped with _*HN*_*qEnA5*^*th*^*-1a* and _*HN*_*qEnA-1*; on chromosome 3, *qhir2* overlapped with _*P2*_*qEnA5*^*th*^*-3b*, _*P2*_*qEnA-3b*, _*P2*_*qEnA-3c*, _*P2*_*qTA-3*, *qhir3* and *ig1* overlapped with _*p2*_*qEnA5*^*th*^*-3a*, _*p2*_*qEnA-3a*, _*HN*_*qEnA-3a*, _*HN*_*qTA-3b*, respectively. 15 QTL for kernel abortion located on chromosome 5 were linked with *qhir6*. That could explain HIR and kernel abortion from in vivo haploid induction were significantly correlated with each other. The regions of chromosome bins 1.04, 3.02, 3.06, and 4.03 were shared by both kernel abortion and haploid production. We also propose that kernel abortion and haploid induction might be controlled by the same genes or one pleiotropic gene. We surveyed QTL regions for potential candidate genes. Candidate genes with important roles in kernel development were located in these regions. For example, the *wrinkled kernel1* (*wrk1*) gene causing small and wrinkled kernels [40,41] is a candidate gene for _*P1*_*qTA-3c* and _*P1*_*qEnA-3c* (bnlg1452-umc1504), sharing the same region in chromosome bin 3.04. The *emb6*, *emb8*, *emb11* (embryo development blocked at coleoptilar stage), *dek8* and *dek10* were also linked with _*P1*_*qTA-4a*, _*P1*_*qEnA-4a*, _*P1*_*qEnA5*^*th*^*-4a* in the region of bnlg1621-umc1317. Clark and Sheridan et al [42,43]. first reported *emb6*, *emb8*, *emb11* and *dek8*, *dek10*, respectively. *Sugars will eventually be exported transporter4c* (*sweet4c*) caused to defective kernels in seed filling [44,45]. It is a candidate gene for QTL _*P1*_*qEnA5*^*th*^*-5a* and _*P1*_*qEnA4*^*th*^*-5a* located on chromosome 5. The *ameiotic1* (*am1*) gene is a candidate for _P1_*qEnA-5c* and _*P1*_*qTA-5c*, located between phi109188 and bnlg1879 on chromosome 5. Cande and Freeling [46] found that this mutant plays an important role in establishing the meiotic cell cycle. *ZmSRS1-2* (*GRMZM2G414043*) is a candidate gene for _*P2*_*qEnA2*^*nd*^*-7*. It is located in bin 7.04 and significantly associated with kernel length [47]. *Ig1-as2 like1* (*ial1*), is very similar to *ig1* encoding a Lateral Organ Boundaries (LOB) domain protein required for embryo sac and leaf development [7]. This gene is a candidate for QTL _*HN*_*qEmA-8*, _*HN*_*qTA-8*, _*P2*_*qTA-8a* and _*ZZ*_*qEnA2*^*nd*^*-8*, located in the same region between markers umc1997 and umc2395 on chromosome 8. Other candidate genes for kernel abortion QTL caused by *in vivo* haploid induction include kernel defective mutant genes: the *defective kernel33* (*dek33*) gene is linked with _*P1*_*qTA-5a*, _*P1*_*qEnA-5a*, _*P1*_*qEnA5*^*th*^*-5a* and _*P1*_*qEnA4*^*th*^*-5a* on chromosome 5. The QTL _*ZZ*_*qEnA3*^*rd*^*-10*, _*zz*_*qEnA5*^*th*^*-10* and _*zz*_*qEnA-10* are co-located with *dek14* on chromosome 10 (Fig 4).

According to the current study, the relationship between haploid induction and kernel abortion suggest that this kind of kernel defective is not only coming from the process of double fertilization, but may also resulting from the mechanism of haploid induction. Tian *et al* [1] observed ovules with an unfertilized egg cell and a fertilized central cell (endosperm) during the process of the haploid induction crosses, which provided direct evidence for the single-fertilization hypothesis and identifying the origins of defective kernels produced in vivo, it was inferred single fertilization and chromosome elimination may lead to formation of defective kernels caused by the inducer pollen. The majority of previous studies have mainly focused on the haploid induction rate improvement but ignored simultaneous effects occurring by haploid induction. Kernel defects present during *in vivo* haploid induction is a key factor affecting haploid production itself. It is different form normal kernel abortion mutations although the phenotype is similar. We used two sets of F_2:3_ families to analyze the different degree of endosperm abortion and embryo abortion and located QTL related with each of them. It would be useful to understand the relationship of haploid induction and embryo abortion in more detail to enhance the efficiency of haploid production.

## Conclusion

In this study, two sets of segregation populations were employed to detect the QTL of different types of abortion kernels during haploid induced by in vivo. Based on the kernel phenotype, different kernel types of embryo abortion and endosperm abortion from 1^st^ to 5^th^ were described firstly. Sixty-two QTL related to the kernel abortion in both populations, most of them related with endosperm abortion, only 3 QTL linked to embryo abortion and located in chromosome 1, 2 and 4, respectively. Seven regions in the chromosomes were shared by 3–4 QTL of different phenotypes at each region in both populations. It was indicated that these loci appeared multiple effects for each locus. Furthermore, through the analysis of the co-localizations of QTL for kernel abortion and the traits related to haploid induction rate (HIR), it was found that nine of twelve QTL related to HIR shared or overlapped with the region of kernel abortions. The total abortion rate (TAR) showed highly significant correlations with HIR. It could be useful to reveal the more genetic basis of HIR and make the doubled haploid breeding with more efficiency in maize breeding practices.
